# The 10th Anniversary of *Bioengineering*: Biochemical Engineering

**DOI:** 10.3390/bioengineering12040378

**Published:** 2025-04-02

**Authors:** Christoph Herwig

**Affiliations:** Lisalis GmbH, Jenbachgasse 73-2, 1130 Vienna, Austria; christoph.herwig@lisalis.at

Biochemical engineering is a multidisciplinary field that utilizes principles of biology, chemistry, and engineering to develop processes and products involving biological materials. It aims to address global challenges related to healthcare, environmental sustainability, energy production, and industrial innovation by harnessing the potential of biological systems and processes. Due to advancements in molecular biology, biotechnology, genomics, computational methods, data science, and digitalization, the scope of biochemical engineering continually expands. As one of the first established sections of the journal *Bioengineering*, “Biochemical Engineering” has been at the forefront of disseminating cutting-edge research and advancements in the field. To commemorate this milestone, we are gratefully setting up this Special Issue.

As you can see from the publications collected in this Special Issue, the potential of biochemical engineering to optimize processes and to provide new products is vast. Although it represents the origin of biochemical engineering, there is still scope for a significant amount of innovation in the area of bioreactor characterization, including the link between hydrodynamics and cellular kinetics [1]. In addition, the process can only be optimized with proper screws, thus the development and optimization of cultivation media is key [2]. Without knowing the liming components of media, we cannot achieve anything.

Large industrial bioreactors were initially used to produce primary and secondary metabolites, such as ethanol and amino acids and antibiotics. This product portfolio has expanded significantly in two directions: (1) We have seen the huge potential of biochemical engineering solutions in the field of sustainable energy [3] and sustainable chemical product production [4], which significantly reduce CO_2_ footprints compared to conventional process routes. (2) Biochemical engineering has enabled the production of novel biopharmaceuticals and represents a step towards provide affordable drugs across the world. However, the field of biopharmaceuticals has reached the point at which economic optimization is crucial for businesses to survive. Therefore, current and future applications of biochemical engineering principles must take place in the area of continuous manufacturing [5].

It can be seen, therefore, that biochemical engineering is an extremely important methodological discipline for the above-mentioned products. We must maintain and enhance the currently available methodological toolset. Thus, even for scholars working in different fields of biotechnological processing, the methods discussed in the publications within this Special Issue represent a very good crossover regarding the processes and systems important across the entire discipline:

- CFD simulations of hydrodynamics;
- Designs of experiments and quality-by-design-related methodologies, ranging from PAT to knowledge management;
- Data science enablers for the acceleration of product life cycles;
- The use of digital twins for the prediction and optimization of bioprocesses;
- End-to-end solutions for achieving intensified bioprocesses and continuous biomanufacturing (CBM).

In the future, we will see further methodological advancements, such as process systems engineering (PSE) principles for enhanced feedback control and the use of ML and AI to robustify and optimize individual unit operations and entire end-to-end bioprocesses. Thus, biochemical engineering is much more than just building bioreactors; it combines a plethora of engineering principles with future data science.
